# Supplementary material for: Plant Functional Dispersion, Vulnerability and Originality Increase Arthropod Functions from a Protected Mountain Mediterranean Area in Spring
Source: Plants (Basel). 2023 Feb 16;12(4):889. doi: 10.3390/plants12040889 (PMC9960503; doi:10.3390/plants12040889)
Supplement: Supplementary file 1 [file plants-12-00889-s001.zip › FigureS1.pdf]

Percentage of ground cover by plant family

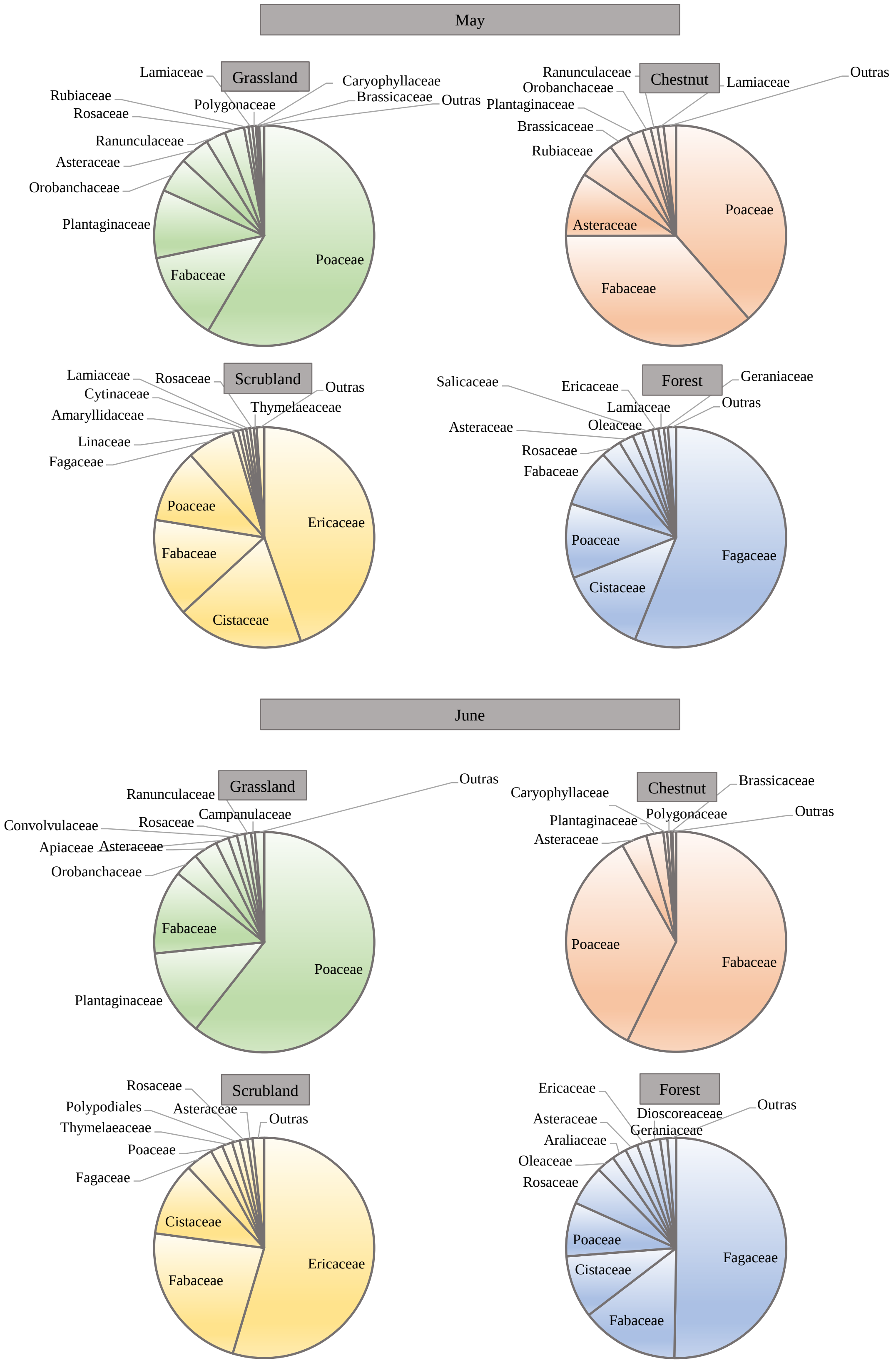

Figure S1. Percentage of ground cover by plant family per habitat and month
