# Supplementary material for: Plant Functional Dispersion, Vulnerability and Originality Increase Arthropod Functions from a Protected Mountain Mediterranean Area in Spring
Source: Plants (Basel). 2023 Feb 16;12(4):889. doi: 10.3390/plants12040889 (PMC9960503; doi:10.3390/plants12040889)
Supplement: Supplementary file 1 [file plants-12-00889-s001.zip › FigureS2.pdf]

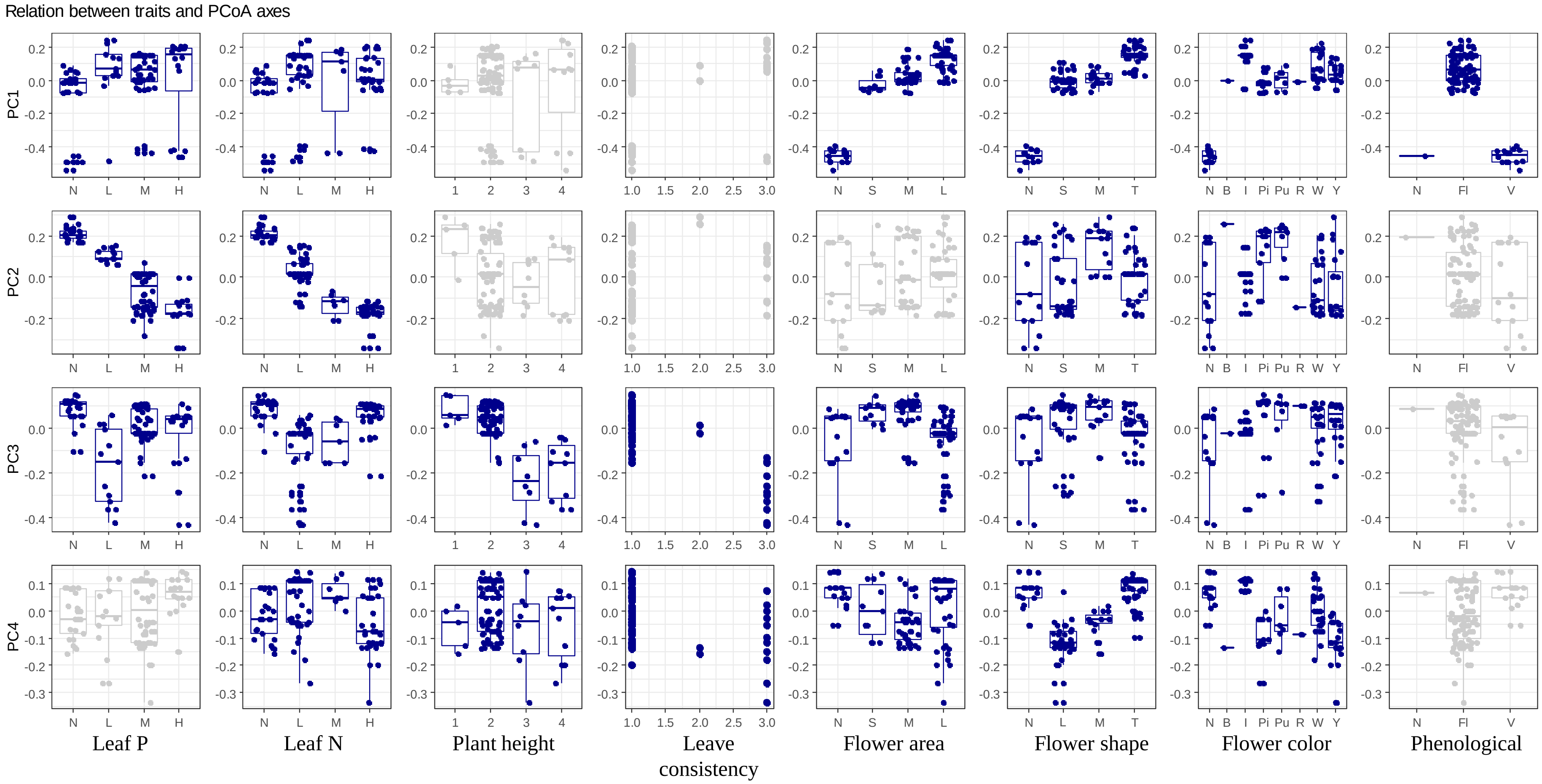

Figure S2. Relation between traits and PCoA axes in May. The significant traits are shown in blue. Leaf P – N: none, L: low, M: medium, H: high. Leaf N – N: none, L: low, M: medium, H: high. Inflorescence area – N: none, S: small, M: medium, L: large. Flower shape: N: none, L: low, M: medium, T: total. Flower color – N: none, B: brown, I: inconspicuous, Pi: Pink, Pu: Purple, R: Red, W: white, Y: Yellow. Phenological: None: none, Fl: Flower, V: vegetative.
