# Supplementary material for: Plant Functional Dispersion, Vulnerability and Originality Increase Arthropod Functions from a Protected Mountain Mediterranean Area in Spring
Source: Plants (Basel). 2023 Feb 16;12(4):889. doi: 10.3390/plants12040889 (PMC9960503; doi:10.3390/plants12040889)
Supplement: Supplementary file 1 [file plants-12-00889-s001.zip › TableS1.pdf]

Table S1. Percentage of ground cover by plant species per habitat and month

Family	Species	May				June			
		Chestnut	Grassland	Scrubland	Forest	Chestnut	Grassland	Scrubland	Forest
Amaryllidaceae	*Allium sphaerocephalon* L.	0	0	1.11	0	0	0.32	0	0.38
Apiaceae	*Achillea millefolium* L.	0	0	0	0	0	2.56	0	0
	Carum verticillatum (L.) W.D.J.Koch	0	0.35	0	0	0	0	0	0
	Galium lucidum subsp. *lucidum* All.	0	0	0	0	0	0	0	0.38
	Torilis arvensis (Huds.) Link	0	0	0	1.36	0	0	0	0
Araliaceae	*Hedera helix sl*	0	0	0.42	0	0	0	0.98	7.51
Asparagaceae	*Muscari comosum* (L.) Mill.	0.24	0	0	0	0	0	0	0
Asteraceae	*Andryala integrifolia* L.	0	0	0	0	0.33	0	1.47	0.38
	Anthemis arvensis L.	2.83	2.07	0.42	0	1.25	0.64	0	0
	Bellis perennis L.	1.00	0.35	0	0	0.50	0	0	0
	Chondrilla juncea L.	0	0	0	0	0.50	0	0	0
	Crepis capillaris (L.) Wallr.	0	0	0	0	0.33	0	0	0
	Crepis vesicaria L.	0.24	0	0	1.36	0	0	0	0
	Filago vulgaris Lam.	0	0	0	0	0.55	0	0	0
	Galactites tomentosus Moench	0	0	0	2.72	0	0	0	2.25
	Hedypnois cretica (L.) Dum.-Courset	1.20	0	0	0	0	0	0	0
	Hypochaeris radicata L.	0	1.24	0	0.27	0.33	0.93	0	0.38
	Leontodon taraxacoides (Vill.) Mérat	0.24	0	0	0	0	0	0	0
	Logfia gallica (L.) Coss. & Germ.	0	0	0	0.27	0	0	0	0
	Senecio vulgaris L.	0	0	0	0	0.33	0	0	0
	Tolpis barbata (L.) Gaertn	0	0	0	0	0	0	0.98	0
Boraginaceae	*Echium plantagineum* L.	0	0	0	0	0	0.32	0	0
	Myosotis discolor Pers.	0.24	0.35	0	0	0	0	0	0
Brassicaceae	*Bunias erucago* L.	3.82	0	0	0	0.66	0	0	0
	Capsella bursa-pastoris (L.) Medik.	0.24	0	0	0	0	0	0	0

Family	Species	May				June			
		Chestnut	Grassland	Scrubland	Forest	Chestnut	Grassland	Scrubland	Forest
	Lepidium heterophyllum Benth.	0	0.52	0	0	0	0.32	0	0
	Raphanus raphanistrum L.	0	0	0	0	0	0.64	0	0
Campanulaceae	*Campanula rapunculus* L.	0	0	0	0	0	0	0.73	0
	Jasione montana L.	0	0	0	0.27	0	0	0.49	0.38
	Linum bienne Mill.	0	0	0	0	0	0.56	0	0
Caryophyllaceae	*Arenaria montana* subsp. *montana* L.	0	0	0	0.27	0	0	0	0.38
	Cerastium brachypetalum Desp. ex Pers.	0.24	0.35	0	0	0.33	0.32	0	0
	Petrorhagia nanteuilii (Burnat) P.W.Ball & Heywood	0	0	0	0	0.44	0	0	0
	Stellaria media (L.) Vill.	0.24	0.35	0	0	0	0	0	0
Cistaceae	*Cistus ladanifer* L.	0	0	7.27	4.08	0	0	3.26	4.50
	Cistus psilosepalus Sweet	0	0	0	0	0	0	0	2.50
	Cistus salviifolius L.	0	0	0	2.38	0	0	5.38	0
	Halimium lasianthum subsp. *lasianthum* (Lam.) Spach	0	0	6.64	1.03	0	0	3.24	1.13
	Helianthemum aegyptiacum (L.) Mill.	0	0	0	1.69	0	0	0	0
Convolvulaceae	*Convolvulus arvensis* L.	0	0	0	0	0.66	1.71	0	0
Crassulaceae	*Sedum forsterianum* Sm.	0	0	0	0	0	0	0	0.38
	Umbilicus rupestris (Salisb.) Dandy	0	0	0	0	0	0	0	0.38
Cytinaceae	*Cytinus hypocistis* (L.) L.	0	0	0.97	0	0	0	0.49	0
Dioscoreaceae	*Tamus communis* L.	0	0	0	0	0	0	0	2.82
Ericaceae	*Erica arborea* L.	0	0	0	1.09	0	0	0	0
	Erica australis L.	0	0	25.84	0	0	0	28.26	3.19
	Erica umbellata L.	0	0	0	0	0	0	4.89	0
Fabaceae	*Coronilla repanda* (Poir.) Guss.	0	0	0	0.27	0	0.64	0	0.75
	Cytisus multiflorus (L'Hér.) Sweet	0	0	2.35	1.52	0	0	6.72	4.22

		May				June			
Family	Species	Chestnut	Grassland	Scrubland	Forest	Chestnut	Grassland	Scrubland	Forest
	Cytisus scoparius subsp. *scoparius* (L.) Link	0	0	1.11	2.72	0	0	6.11	0
	Cytisus sp	0	0	0	0	0	0	0	6.57
	Cytisus striatus (Hill) Rothm.	0	0	0	0	0	0	0	3.75
	Genista florida L.	0	0	0	4.76	0	0	0	3.75
	Genista hystrix Lange	0	0	0	0	0	0	0.98	4.69
	Lathyrus cicera L.	0.72	0	0.42	0	0.33	0	0	0
	Lotus corniculatus L.	0.67	0	0	0.38	1.66	0	0.49	0.38
	Lotus pedunculatus Cav.	0	0	0	0	0.33	0	0	0
	Medicago polymorpha L.	1.20	0	0	0	0.33	0	0	0
	Ononis spinosa L.	0	0	0	0	0.33	0	0	0
	Ornithopus compressus L.	0.68	0	0	0.27	1.22	0.32	0	0
	Ornithopus perpusillus L.	0.72	0	0	0.27	1.00	0.32	0	0.38
	Pterospartum tridentatum (L.) Willk.	0	0	7.01	0	0	0	8.37	0
	Trifolium arvense L.	0	0	0	0	0	0	0.49	0
	Trifolium campestre Schreb.	1.20	0	0.42	0	0.66	0.32	0	0.38
	Trifolium cernuum Brot.	0	0	0	0	0.33	1.60	0	0
	Trifolium dubium Sibth.	0.24	2.35	0	0	0	0	0	0
	Trifolium glomeratum L.	7.17	0	0	0	1.00	0	0.49	0
	Trifolium incarnatum L.	1.20	1.73	0	0	0	0.64	0	0
	Trifolium pratense L.	5.96	0.35	0	0	3.49	1.46	0	0
	Trifolium repens L.	6.57	4.68	0	0	16.61	2.96	0	0
	Trifolium resupinatum L.	0.24	0	0	0	0.66	1.76	0	0
	Trifolium stellatum L.	0	0	0	0	16.61	0	0	0
	Trifolium striatum L.	0	0	0	0	0	0.85	0	0
	Trifolium strictum L.	0	0.92	0	0	0	0	0	0
	Trifolium subterraneum L.	10.16	0	0	0	0	0	0	0

Family	Species	May				June			
		Chestnut	Grassland	Scrubland	Forest	Chestnut	Grassland	Scrubland	Forest
	Trifolium vesiculosum Savi	0	0	0	0	1.66	0	0	0
	Vicia angustifolia L.	0.24	0.78	0.42	0.27	0.50	0.32	0	0.38
	Vicia hirsuta (L.) Gray	2.51	0	0	0	0.33	0	0	0
	Vicia lutea L.	0.24	0	0	0	0	0	0	0
	Vicia villosa Roth	0	0	0	0	0	0.32	0	0
Fagaceae	*Quercus pyrenaica* Willd.	0	0	3.03	0	0	0	1.41	0
	Quercus rotundifolia Lam.	0	0	3.07	17.24	0	0	3.26	14.70
Geraniaceae	*Erodium cicutarium* (L.) L'Hér.	0	0.35	0	0	0	0.64	0	0
	Erodium sp.	0.24	0	0	0	0	0	0	0
	Geranium dissectum L.	0	0.35	0	0	0	0.32	0	0
	Geranium lucidum L.	0	0	0	0	0	0	0	0.56
	Geranium molle L.	0.24	0	0.42	0.73	0	0	0	0
	Geranium robertianum L.	0	0	0	0	0	0	0	1.31
Hypericaceae	*Hypericum perforatum* L.	0.24	0	0.83	0	0.66	0.32	2.45	0
Lamiaceae	*Acinos alpinus* (L.) Moench	0	1.04	0	0	0	0	0	0
	Clinopodium vulgare L.	0	0	0	0	0.33	0	0.49	0
	Lavandula pedunculata (Mill.) Cav.	0	0	2.08	1.00	0	0	0.49	1.88
	Mentha suaveolens Ehrh.	0	0.35	0	0	0	0.32	0	0
	Stachys arvensis (L.) L.	1.31	0	0	0	0	0	0	0
	Thymus mastichina L.	0	0	0.83	0	0	0	0.98	0.38
Linaceae	*Linum bienne* Mill.	0	0.35	4.15	0	0	0	0	0
Oleaceae	*Fraxinus angustifolia* Vahl	0	0	0.42	5.44	0	0	0.98	4.69
Orchidaceae	*Serapias lingua* L.	0	0.35	0	0	0	0	0	0
Orobanchaceae	*Orobanche rapum-genistae* Thuill.	0	0	0.42	0	0	0	0.49	0
	Parentucellia latifolia (L.) Caruel	1.79	0	0	0	0	0	0	0
	Rhinanthus minor L.	0	4.98	0	0	0	4.80	0	0
Plantaginaceae	*Anarrhinum bellidifolium* (L.) Willd.	0.24	0	0	0	0.33	0	0.49	0

Family	Species	May				June			
		Chestnut	Grassland	Scrubland	Forest	Chestnut	Grassland	Scrubland	Forest
	Digitalis purpurea L.	0	0	0	0	0	0.32	0	0
	Plantago lanceolata L.	1.34	4.84	0	0	1.83	4.19	0	0
	Reseda luteola L.	0	0	0	0	0	0.32	0	0
Poaceae	*Aegilops triuncialis* L.	0	0	0	0	6.65	0	0	0
	Agrostis castellana Boiss. & Reut.	0	0	0	0	0.33	11.74	0	0
	Anthoxanthum amarum Brot.	5.98	0.35	0	5.44	6.65	5.87	0	3.75
	Anthoxanthum aristatum subsp. *aristatum* Boiss.	0	0	0	13.61	0	2.13	0	0
	Anthoxanthum odoratum L.	0	0.81	0	0	0	0	0	0
	Arrhenatherum elatius (L.) J.Presl & C.Presl	4.78	0.46	1.25	0	2.99	4.80	0	3.57
	Avena barbata Link	1.20	1.50	0	0.27	0.50	1.60	0	7.51
	Avenula sulcata (Boiss.) Dumort.	0	2.59	0	5.44	0	0	0	0
	Briza maxima L.	0	0	0	0	0	0	0.98	0
	Briza minor L.	0	0.35	0	0	0	0.32	0	0
	Bromus diandrus Roth	1.79	8.64	0	4.76	1.66	6.40	0	0
	Bromus hordeaceus L.	0.88	3.55	0	0	1.33	0.43	0	0
	Bromus madritensis L.	0	5.36	0	0	3.32	5.55	0	0
	Bromus sp	3.59	0	15.23	1.50	0	0	0	0
	Bromus sterilis L.	2.99	0	0	0	0	0	0	0
	Bromus tectorum L.	2.99	4.32	0	0	0	0	0	0
	Cynosurus cristatus L.	0	4.56	0	0	4.54	3.73	0	0
	Cynosurus echinatus L.	2.39	0	0	0	0.50	0	0	0.75
	Dactylis glomerata L.	0.84	1.73	4.15	0.82	0.80	0.48	2.77	0.75
	Festuca arundinacea Schreb.	0	0	0	0	0.33	4.00	0	0
	Holcus lanatus L.	0.48	5.67	0	0	0.66	8.00	0	0
	Hordeum murinum L.	0	0	0	0	0.66	0	0	0

Family	Species	May Chestnut	May Grassland	May Scrubland	May Forest	June Chestnut	June Grassland	June Scrubland	June Forest
	Lolium rigidum subsp. *rigidum* Gaudin	4.01	1.61	0	0	3.54	6.19	0	0
	Molineriella laevis (Brot.) Rouy	0.84	0	4.15	0	0	0	0	0
	Poaceae	0	17.28	0	0	0.60	0	0	0
	Trisetaria flavescens subsp. flavescens (L.) Maire	0	0	0	0	0	3.20	0	0
	Vulpia sp.	5.68	3.46	0	1.36	6.65	1.12	0	0
Polygalaceae	*Polygala microphylla* L.	0	0	0	0	0	0	0.49	0
	Polygala vulgaris L.	0	0	0.42	0	0	0	0	0
	Rumex acetosa subsp. *acetosa* L.	0	0.69	0	0	0	0	0	0
	Rumex acetosella subsp. *angiocarpus* (Murb.) Murb.	0	1.73	0	0	0.66	0.64	0	0
	Rumex bucephalophorus L.	0.29	0	0	0	0	0	0	0
	Rumex conglomeratus Murray	0.24	0	0	0	0.50	0	0	0.38
Polypodiales	NI	0	0	0	0	0	0	1.96	0
	Pteridium aquilinum subsp. *aquilinum*	0	0	0.42	0	0	0	0	0
Ranunculaceae	*Ranunculus bulbosus* L.	0.96	1.57	0	0	0.33	0.96	0	0
Resedaceae	*Reseda luteola* L.	0	0.52	0	0.27	0	0	0	0
	Sesamoides purpurascens (L.) G.López	0.24	0	0	0	0	0	0	0
Rosaceae	*Rosa* sp.	0	0	1.45	2.72	0	0	4.89	6.88
	Rubus ulmifolius var. *ulmifolius* Schott	0	0	0	5.44	0	0	0	0
	*Sanguisorba verrucos*a (Link ex G.Don) Ces.	0	2.76	0	0	0.33	1.12	0.98	0
Rubiaceae	*Galium aparine* L.	0	0	0	0.95	0	0	0	0
	Galium lucidum subsp. *lucidum* All.	0	0	0	0	0	0	0	0.75
	Galium parisiense L.	0	1.04	0.83	0	0	0	0	0

Family	Species	May				June			
		Chestnut	Grassland	Scrubland	Forest	Chestnut	Grassland	Scrubland	Forest
	Galium verum subsp. *verum* L.	0	0	0	0	0	0.64	0	0
	Sherardia arvensis L.	4.00	0.52	0	0	0	0	0	0
Salicaceae	*Populus nigra* L.	0	0	0	5.44	0	0	0	0
Saxifragaceae	*Saxifraga granulata* L.	0.24	0	0	0	0	0	0	0
Thymelaeaceae	*Daphne gnidium* L	0	0	2.08	0.27	0	0	2.61	0
Xanthorrhoeaceae	*Simethis mattiazzi* (Vand.) Sacc.	0	0	0.42	0	0	0	0.49	0
