# Supplementary material for: Plant Functional Dispersion, Vulnerability and Originality Increase Arthropod Functions from a Protected Mountain Mediterranean Area in Spring
Source: Plants (Basel). 2023 Feb 16;12(4):889. doi: 10.3390/plants12040889 (PMC9960503; doi:10.3390/plants12040889)
Supplement: Supplementary file 1 [file plants-12-00889-s001.zip › TableS2.pdf]

Table S2. Relation between traits and PCoA axes: trait, principal component (PC), test, statistic name, statistic value and p-value

May						June					
**trait**	**axis**	**test**	**statistic**	**value**	**p-value**	**trait**	**axis**	**test**	**statistic**	**value**	**p-value**
**leaf P**	PC1	Kruskal-Wallis	eta2	0.202	< 0.001	**leaf P**	PC2	Kruskal-Wallis	eta2	0.690	< 0.001
**leaf P**	PC2	Kruskal-Wallis	eta2	0.749	< 0.001	**leaf P**	PC3	Kruskal-Wallis	eta2	0.429	< 0.001
**leaf P**	PC3	Kruskal-Wallis	eta2	0.330	< 0.001	**leaf N**	PC1	Kruskal-Wallis	eta2	0.151	< 0.001
**leaf P**	PC4	Kruskal-Wallis	eta2	0.104	0.003	**leaf N**	PC2	Kruskal-Wallis	eta2	0.883	< 0.001
**leaf N**	PC1	Kruskal-Wallis	eta2	0.178	< 0.001	**leaf N**	PC3	Kruskal-Wallis	eta2	0.547	< 0.001
**leaf N**	PC2	Kruskal-Wallis	eta2	0.868	< 0.001	**leaf N**	PC4	Kruskal-Wallis	eta2	0.067	0.016
**leaf N**	PC3	Kruskal-Wallis	eta2	0.520	< 0.001	**Plant height**	PC1	Kruskal-Wallis	eta2	0.048	0.042
**leaf N**	PC4	Kruskal-Wallis	eta2	0.119	0.001	**Plant height**	PC3	Kruskal-Wallis	eta2	0.452	< 0.001
**Plant height**	PC3	Kruskal-Wallis	eta2	0.386	< 0.001	**Plant height**	PC4	Kruskal-Wallis	eta2	0.095	0.004
**Leave consistency**	PC3	Linear Model	r2	0.704	< 0.001	**Leave consistency**	PC3	Linear Model	r2	0.546	< 0.001
**Inflorescence area**	PC1	Kruskal-Wallis	eta2	0.663	< 0.001	**Leave consistency**	PC4	Linear Model	r2	0.134	< 0.001
**Inflorescence area**	PC3	Kruskal-Wallis	eta2	0.409	< 0.001	**Inflorescence area**	PC1	Kruskal-Wallis	eta2	0.803	< 0.001
**Inflorescence area**	PC4	Kruskal-Wallis	eta2	0.049	0.043	**Inflorescence area**	PC3	Kruskal-Wallis	eta2	0.235	< 0.001
**Flower shape**	PC1	Kruskal-Wallis	eta2	0.717	< 0.001	**Inflorescence area**	PC4	Kruskal-Wallis	eta2	0.256	< 0.001
**Flower shape**	PC2	Kruskal-Wallis	eta2	0.123	0.001	**Flower shape**	PC1	Kruskal-Wallis	eta2	0.882	< 0.001
**Flower shape**	PC3	Kruskal-Wallis	eta2	0.114	0.002	**Flower shape**	PC2	Kruskal-Wallis	eta2	0.175	< 0.001
**Flower shape**	PC4	Kruskal-Wallis	eta2	0.491	< 0.001	**Flower shape**	PC3	Kruskal-Wallis	eta2	0.095	0.004
**Flower color**	PC1	Kruskal-Wallis	eta2	0.406	< 0.001	**Flower shape**	PC4	Kruskal-Wallis	eta2	0.608	< 0.001
**Flower color**	PC2	Kruskal-Wallis	eta2	0.178	0.001	**Flower color**	PC1	Kruskal-Wallis	eta2	0.735	< 0.001
**Flower color**	PC3	Kruskal-Wallis	eta2	0.104	0.010	**Flower color**	PC3	Kruskal-Wallis	eta2	0.114	0.008
**Flower color**	PC4	Kruskal-Wallis	eta2	0.509	< 0.001	**Flower color**	PC4	Kruskal-Wallis	eta2	0.776	< 0.001
**Phenological**	PC1	Kruskal-Wallis	eta2	0.343	< 0.001	**Phenological**	PC1	Kruskal-Wallis	eta2	0.594	< 0.001
