# Supplementary material for: Plant Functional Dispersion, Vulnerability and Originality Increase Arthropod Functions from a Protected Mountain Mediterranean Area in Spring
Source: Plants (Basel). 2023 Feb 16;12(4):889. doi: 10.3390/plants12040889 (PMC9960503; doi:10.3390/plants12040889)
Supplement: Supplementary file 1 [file plants-12-00889-s001.zip › TableS3.pdf]

Table S3. GLM and LM outputs for the estimated regression parameters and standard errors of the response of (i) taxonomic and (ii) functional plant diversity indexes to the habitat type (grassland, chestnut, scrubland, oak forest).

(i) Taxonomic diversity

May

Richness - Trich				
**Generalized Linear model: glm(Trich~Environment, Family ="poisson")**				
	**Estimate**	**SE**	**Statistic**	**p-value**
Grassland	2.944	0.178	16.539	0.000
Chestnut	0.147	0.248	0.591	0.555
Scrubland	-0.547	0.270	-2.023	0.043
Forest	-0.419	0.265	-1.580	0.114

June

Richness - Trich				
**Generalized Linear model: glm.nb(Trich~Environment)**				
	**Estimate**	**SE**	**Statistic**	**p-value**
Grassland	2.970	0.205	14.492	0.000
Chestnut	0.000	0.290	0.000	1.000
Scrubland	-0.465	0.303	-1.536	0.125
Forest	-0.405	0.301	-1.348	0.178

May

Shannon diversity Index -SDI				
**Generalized Linear model: glm(SDI~Environment, family="Gamma")**				
	**Estimate**	**SE**	**Statistic**	**p-value**
Grassland	0.438	0.079	5.584	0.000
Chestnut	-0.015	0.109	-0.140	0.891
Scrubland	0.260	0.148	1.763	0.103
Forest	0.151	0.131	1.145	0.274

June

Shannon diversity Index - SDI				
**Linear model: glm(SDI~Environment, family="Gamma")**				
	**Estimate**	**SE**	**Statistic**	**p-value**
Grassland	0.427	0.075	5.710	0.000
Chestnut	0.036	0.110	0.323	0.752
Scrubland	0.194	0.132	1.466	0.168
Forest	0.127	0.123	1.034	0.321

(ii) Functional diversity

May

Functional dispersion - Fdis				
**Generalized Linear model: glm(Fdis~Environment, Family ="Gamma")**				
	**Estimate**	**SE**	**Statistic**	**p-value**
Grassland	2.307	0.312	7.391	0.000
Chestnut	-0.096	0.432	-0.222	0.828
Scrubland	-0.115	0.431	-0.267	0.794
Forest	-0.407	0.404	-1.007	0.334

June

Functional dispersion - Fdis				
**Generalized Linear model: glm(Fdis~Environment, Family ="Gamma")**				
	**Estimate**	**SE**	**Statistic**	**p-value**
Grassland	2.901	0.409	7.085	0.000
Chestnut	0.215	0.601	0.358	0.726
Scrubland	-1.322	0.466	-2.837	0.015
Forest	-1.608	0.448	-3.587	0.004

May

Functional richness - Fric				
**Generalized Linear model: glm(Fric~Environment, Family ="Gamma")**				
	**Estimate**	**SE**	**Statistic**	**p-value**
Grassland	51.540	34.968	1.474	0.166
Chestnut	-15.289	42.751	-0.358	0.727
Scrubland	-43.663	35.374	-1.234	0.241
Forest	-41.116	35.676	-1.152	0.272

June

Functional richness - Fric				
**Generalized Linear model: glm(Fric~Environment, Family ="Gamma")**				
	**Estimate**	**SE**	**Statistic**	**p-value**
Grassland	8.583	2.880	2.980	0.011
Chestnut	5.100	5.420	0.941	0.365
Scrubland	-6.118	2.997	-2.041	0.064
Forest	-6.224	2.987	-2.084	0.059

Functional divergence - Fdiv				
**Generalized Linear model: glm(Fdiv~Environment, Family ="Gamma")**				
	**Estimate**	**SE**	**Statistic**	**p-value**
Grassland	1.134	0.052	21.705	0.000
Chestnut	0.068	0.076	0.888	0.392
Scrubland	0.027	0.075	0.359	0.726
Forest	-0.016	0.073	-0.216	0.833

Functional eveness - Feve				
**Generalized Linear model: glm(Feve~Environment, Family ="Gamma")**				
	**Estimate**	**SE**	**Statistic**	**p-value**
Grassland	2.069	0.144	14.404	0.000
Chestnut	0.195	0.213	0.914	0.379
Scrubland	0.061	0.206	0.297	0.772
Forest	-0.382	0.185	-2.060	0.062

Functional specialization - Fspe				
**Generalized Linear model: glm(Fspe~Environment, Family ="Gamma")**				
	**Estimate**	**SE**	**Statistic**	**p-value**
Grassland	3.252	0.258	12.582	0.000
Chestnut	0.076	0.370	0.205	0.841
Scrubland	-1.603	0.290	-5.530	0.000
Forest	-1.505	0.293	-5.131	0.000

Functional originality - Fori				
**Generalized Linear model: glm(Fori~Environment, Family ="Gamma")**				
	**Estimate**	**SE**	**Statistic**	**p-value**
Grassland	6.737	2.097	3.213	0.007
Chestnut	6.131	4.521	1.356	0.200
Scrubland	-4.149	2.247	-1.847	0.090
Forest	7.101	4.791	1.482	0.164

Number of functional entities - Fnfe				
**Generalized Linear model: glm(Fnfe~Environment, Family ="Gamma")**				
	**Estimate**	**SE**	**Statistic**	**p-value**
Grassland	2.546	0.174	14.589	0.000
Chestnut	0.195	0.240	0.815	0.415
Scrubland	-0.243	0.257	-0.944	0.345
Forest	-0.171	0.254	-0.672	0.502

Functional divergence - Fdiv				
**Generalized Linear model: glm(Fdiv~Environment, Family ="Gamma")**				
	**Estimate**	**SE**	**Statistic**	**p-value**
Grassland	1.171	0.086	13.572	0.000
Chestnut	0.205	0.133	1.537	0.150
Scrubland	-0.047	0.120	-0.394	0.701
Forest	-0.076	0.118	-0.647	0.530

Functional eveness -Feve				
**Generalized Linear model: glm(Feve~Environment, Family ="Gamma")**				
	**Estimate**	**SE**	**Statistic**	**p-value**
Grassland	2.479	0.284	8.737	0.000
Chestnut	0.350	0.430	0.813	0.432
Scrubland	-0.143	0.390	-0.367	0.720
Forest	-0.692	0.350	-1.979	0.071

Functional specialization -Fspe				
**Generalized Linear model: glm(Fspe~Environment, Family ="Gamma")**				
	**Estimate**	**SE**	**Statistic**	**p-value**
Grassland	2.473	0.162	15.238	0.000
Chestnut	0.280	0.243	1.152	0.272
Scrubland	-1.204	0.182	-6.598	0.000
Forest	-1.102	0.186	-5.938	0.000

Functional originality - Fori				
**Generalized Linear model: glm(Fori~Environment, Family ="Gamma")**				
	**Estimate**	**SE**	**Statistic**	**p-value**
Grassland	9.505	3.573	2.660	0.021
Chestnut	15.542	10.071	1.543	0.149
Scrubland	-5.542	3.872	-1.431	0.178
Forest	-2.372	4.468	-0.531	0.605

Number of functional entities - Fnfe				
**Generalized Linear model: glm(Fnfe~Environment, Family ="Gamma")**				
	**Estimate**	**SE**	**Statistic**	**p-value**
Grassland	2.603	0.205	12.684	0.000
Chestnut	-0.139	0.295	-0.471	0.638
Scrubland	-0.275	0.300	-0.918	0.359
Forest	-0.160	0.296	-0.542	0.588

Functional Redundancy - Fred				
Generalized Linear model: glm(Fred~Environment, Family ="Gamma")				
	**Estimate**	**SE**	**Statistic**	**p-value**
Grassland	0.668	0.027	24.317	0.000
Chestnut	0.036	0.040	0.900	0.386
Scrubland	0.271	0.047	5.717	0.000
Forest	0.207	0.045	4.574	0.001

Functional Redundancy - Fred				
Generalized Linear model: glm(Fred~Environment, Family ="Gamma")				
	**Estimate**	**SE**	**Statistic**	**p-value**
Grassland	0.705	0.059	11.982	0.000
Chestnut	-0.123	0.076	-1.614	0.132
Scrubland	0.108	0.090	1.200	0.253
Forest	0.174	0.094	1.850	0.089

Functional Vulnerability - Fvuln				
Generalized Linear model: glm(Fvuln~Environment, Family ="Gamma")				
	**Estimate**	**SE**	**Statistic**	**p-value**
Grassland	1.153	0.041	28.074	0.000
Chestnut	0.111	0.061	1.820	0.094
Scrubland	-0.099	0.056	-1.779	0.101
Forest	-0.053	0.057	-0.938	0.367

Functional Vulnerability - Fvuln				
Generalized Linear model: glm(Fvuln~Environment, Family ="Gamma")				
	**Estimate**	**SE**	**Statistic**	**p-value**
Grassland	1.158	0.063	18.436	0.000
Chestnut	0.113	0.093	1.213	0.248
Scrubland	0.044	0.091	0.483	0.637
Forest	-0.038	0.087	-0.432	0.674
