# Supplementary material for: Plant Functional Dispersion, Vulnerability and Originality Increase Arthropod Functions from a Protected Mountain Mediterranean Area in Spring
Source: Plants (Basel). 2023 Feb 16;12(4):889. doi: 10.3390/plants12040889 (PMC9960503; doi:10.3390/plants12040889)
Supplement: Supplementary file 1 [file plants-12-00889-s001.zip › TableS4.pdf]

Table S4. The leaf nitrogen (leaf-N) and phosphorous (leaf-P) composition (mg g $^{-1}$). Mean values – for species when possible, or for plant family – from the literature were used [1-5] [References 31 to 35 in reference list of the manuscript]. For species/families with no information "Not Available" (NA) was introduced in the data matrix. The values were ranked as follows: for N – low (< 19 mg g $^{-1}$), medium (> 19 and < 23 mg g $^{-1}$), high (>23 mg g $^{-1}$), for P – low (< 1.1mg g $^{-1}$), medium (> 1.1 and < 1.7 mg g $^{-1}$), high (> 1.7 mg g $^{-1}$).

Family	Species	leaf-N	leaf-P
Amaranthaceae	*Chenopodium* sp	NA	NA
Amaryllidaceae	*Allium sphaerocephalon* L.	NA	NA
Apiaceae	spp.	high	high
Araliaceae	*Hedera helix* sl	low	high
Asparagaceae	*Muscari comosum* (L.) Mill.	low	low
Asteraceae	spp.	low	medium
Boraginaceae	*Echium plantagineum* L.	high	high
	Myosotis discolor Pers.	NA	NA
Brassicaceae	spp.	NA	NA
Campanulaceae	spp.	NA	NA
Caryophyllaceae	spp.	medium	NA
Cistaceae	*Cistus ladanifer* L	low	low
	Cistus psilosepalus Sweet	low	low
	Cistus salviifolius L.	medium	NA
	Halimium lasianthum subsp. *lasianthum* (Lam.) Spach	low	low
	Helianthemum aegyptiacum (L.) Mill.	low	low
Convolvulaceae	*Convolvulus arvensis* L.	NA	NA
Crassulaceae	spp.	NA	NA
Cytinaceae	*Cytinus hypocistis* (L.) L.	NA	NA
Dioscoreaceae	*Tamus communis* L.	NA	NA
Ericaceae	spp.	low	low
Euphorbiaceae	*Euphorbia* sp.	low	medium
Fabaceae	spp.	high	medium
Fagaceae	spp.	low	low
Geraniaceae	spp.	NA	NA
Hypericaceae	*Hypericum perforatum* L.	NA	NA
Lamiaceae	*Acinos alpinus* (L.) Moench	NA	NA
	Clinopodium vulgare L.	low	high
	Lavandula pedunculata (Mill.) Cav.	low	high
	Mentha suaveolens Ehrh.	NA	NA
	Stachys arvensis (L.) L.	NA	NA
	Thymus mastichina L.	low	high
Linaceae	*Linum bienne* Mill.	NA	NA
Lythraceae	*Lythrum salicaria* L.	NA	NA
Oleaceae	*Fraxinus angustifolia* Vahl	medium	NA
Orchidaceae	*Serapias lingua* L.	NA	NA
Orobanchaceae	spp.	NA	NA
Plantaginaceae	*Anarrhinum bellidifolium* (L.) Willd.	NA	NA
	Digitalis purpurea L.	low	low

Family	Species	leaf-N	leaf-P
	Plantago lanceolata L.	high	medium
Poaceae	spp.	low	medium
Polygalaceae	spp.	NA	NA
Polygonaceae	spp.	high	high
Ranunculaceae	*Ranunculus bulbosus* L.	high	high
Resedaceae	spp.	high	high
Rosaceae	*Crataegus monogyna* Jacq.	low	low
	Prunus avium L.	medium	NA
	Rosa sp.	low	low
	Rubus sp.	medium	NA
	Rubus ulmifolius var. *ulmifolius* Schott	medium	NA
	Sanguisorba verrucosa (Link ex G.Don) Ces.	medium	NA
Rubiaceae	spp.	low	low
Salicaceae	*Populus nigra* L.	NA	NA
Saxifragaceae	*Saxifraga granulata* L.	high	high
Thymelaeaceae	*Daphne gnidium* L	low	low
Ulmaceae	*Ulmus minor* Mill.	high	medium
Xanthorrhoeaceae	*Simethis mattiazzi* (Vand.) Sacc.	high	high

References

1. Margaris, N.S.; Adamandiadou, S.; Siafaca, L.; Diamantopoulos, J. Nitrogen and phosphorus content in plant species of Mediterranean ecosystems in Greece. *Vegetatio.* **1984**, *55*, 29–35. https://doi.org/10.1007/BF00039978
2. Badre, B.; Nobelis, P.; Trémolières. Quantitative study and modelling of the litter decomposition in a European alluvial forest. Is there an influence of overstorey tree species on the decomposition of ivy litter (*Hedera helix* L.). *Acta Oecol.* **1998**, *19*, 491–500. https://doi.org/10.1016/S1146-609X(99)80003-4
3. Tian, D.; Yan, Z.; Ma, S.; Ding, Y.; Luo, Y.; Chen, Y.; Du, E.; Han, W.; Kovacs, D.E.; Shen, H.; Hu, H, Kattge, J.; Schmid, B.; Fang, J. Family-level leaf nitrogen and phosphorus stoichiometry of global terrestrial plants. *Sci. China Life Sci.* **2019**, *8*, 1047–1057. https://doi.org/10.1007/s11427-019-9584-1
4. Mohammadzadeh, S.; Pirzad, A. Biochemical responses of mycorrhizal-inoculated Lamiaceae (Lavender, Rosemary and Thyme) plants to drought: a field study. *J. Soil Sci. Plant Nutr.* **2021**, *67*, 41–49. https://doi.org/10.1080/00380768.2020.1851144
5. Navarro, T.; Hidalgo-Triana, N. Variations in leaf traits modulate plant vegetative and reproductive phenological sequencing across arid Mediterranean shrublands. *Front. Plant Sci.* **2021**, *12*, 708367. https://doi.org/10.3389/fpls.2021.708367
